# High cardiac vagal control is related to better subjective and objective sleep quality

**DOI:** 10.1016/j.biopsycho.2015.02.004

**Published:** 2015-03

**Authors:** Gabriela G. Werner, Brett Q. Ford, Iris B. Mauss, Manuel Schabus, Jens Blechert, Frank H. Wilhelm

**Affiliations:** a Clinical Stress and Emotion Lab, Division of Clinical Psychology, Psychotherapy, and Health Psychology, Department of Psychology, University of Salzburg (Study Institution), Salzburg, Austria; b Laboratory for Sleep, Cognition and Consciousness Research, Division of Biological Psychology, Department of Psychology, University of Salzburg, Salzburg, Austria; c Centre for Cognitive Neuroscience, University of Salzburg, Salzburg, Austria; d Emotion & Emotion Regulation Lab, Department of Psychology, University of California, Berkeley, CA, United States

**Keywords:** Parasympathetic nervous system, Heart rate variability, Respiratory sinus arrhythmia, Sleep quality, Polysomnography

## Abstract

Cardiac vagal control (CVC) has been linked to both physical and mental health. One critical aspect of health, that has not received much attention, is sleep. We hypothesized that adults with higher CVC – operationalized by high-frequency heart rate variability (HF-HRV) – will exhibit better sleep quality assessed both subjectively (i.e., with Pittsburgh Sleep Quality Index) and objectively (i.e., with polysomnography). HF-HRV was measured in 29 healthy young women during an extended neutral film clip. Participants then underwent full polysomnography to obtain objective measures of sleep quality and HF-HRV during a night of sleep. As expected, higher resting HF-HRV was associated with higher subjective and objective sleep quality (i.e., shorter sleep latency and fewer arousals). HF-HRV during sleep (overall or separated by sleep phases) showed less consistent relationships with sleep quality. These findings indicate that high waking CVC may be a key predictor of healthy sleep.

## Highlights

• Cardiac vagal control (CVC) was measured during an extended standardized baseline.
• Subjective and polysomnographic variables of sleep quality were assessed.
• Higher CVC was found to be associated with better subjective and objective sleep quality.

## 1 Introduction

The parasympathetic nervous system is the branch of the autonomic nervous system responsible for key restorative processes like “resting and digesting” (e.g., Sherwood, 2010). Effective functioning of this system has been linked with stronger phasic activity of vagus nerve efferent activity to the sino-atrial node of the heart, often termed *cardiac vagal control* (CVC). CVC can be measured noninvasively by quantification of respiratory sinus arrhythmia, i.e., the rhythmic oscillation in heart rate linked to breathing frequency (Grossman & Taylor, 2007). Greater CVC during resting baseline assessments at wake (CVC_wake_) has been associated with both better physical and mental health (Beauchaine, 2001; Porges, 2007; Thayer & Lane, 2007). One particularly critical aspect of physical and mental health is sleep (Buysse, 2014), raising the possibility that CVC_wake_ would be positively linked with sleep quality. Yet, the links between CVC_wake_ and sleep have not received much attention. Therefore, we tested in healthy adults whether individual differences in CVC_wake_ are linked to sleep quality, as assessed with subjective (i.e., self-report questionnaire) and objective measures (i.e., full polysomnography).

### 1.1 CVC and health

Higher CVC_wake_ has been reliably linked with a range of positive physical health outcomes, including better cardiovascular health (e.g., Giese-Davis et al., 2015; Thayer & Lane, 2007) as well as positive mental health outcomes, including greater subjective well-being (e.g., Beauchaine, 2001; Geisler, Vennewald, Kubiak, & Weber, 2010). While multiple theoretical models have been proposed to account for the positive link between CVC_wake_ and health, most agree that CVC is a marker of processes involved in the regulation of arousal. In this view, the parasympathetic system exerts an inhibitory influence via the vagus nerve, which promotes calm states by actively reducing autonomic arousal (e.g., Berntson et al., 1997; Grossman & Taylor, 2007; Porges, 2007; Thayer & Lane, 2009). One state characterized by lower arousal is sleep, as sleep can only occur when arousal is substantially reduced (Dahl, 1996). Given that CVC_wake_ is thought to be crucially involved in regulating arousal, further supported by links between reduced arousal and increases in CVC_wake_ (e.g., during mediation; Delgado et al., 2010), and given that sleep relies on low arousal, one might expect that sleep would also show a relationship with CVC_wake_.

### 1.2 CVC and sleep

A variety of research has linked CVC and sleep. It has been shown that CVC increases in anticipation of sleep onset (e.g., Burgess, Trinder, Kim, & Luke, 1997) and remains enhanced during sleep (Stein & Pu, 2012; Trinder et al., 2001). Emerging evidence also supports a link between higher CVC_wake_ and better subjective sleep quality in specific populations like children or clinical samples (e.g., El-Sheikh, Erath, & Bagley, 2013; Fang, Huang, Yang, & Tsai, 2008; Hovland et al., 2013; Yang et al., 2011) as well as in healthy adults in the context of daily stressors (Jackowska, Dockray, Endrighi, Hendrickx, & Steptoe, 2012; Kageyama et al., 1998). Only one study measured objective sleep quality and found that higher levels of CVC_wake_ were associated with deeper sleep (i.e., delta power) in participants with alcohol-dependency (e.g., Irwin, Valladares, Motivala, Thayer, & Ehlers, 2006).

Research assessing links between CVC_wake_ and sleep, however, may not speak to the links between CVC during sleep (CVC_sleep_) and sleep. Studies linking CVC_sleep_ (sometimes but not often separated for each sleep stage) and sleep quality showed, for example, reduced levels of CVC_sleep_ in patients with different sleep disorders, such as primary insomnia or in patients with chronic fatigue syndrome (e.g., Burton, Rahman, Kadota, Lloyd, & Vollmer-Conna, 2010; Stein & Pu, 2012; Tobaldini et al., 2013). A few studies also found a link between higher levels of CVC_sleep_ and better subjective sleep quality (e.g., Yang et al., 2011) in patient groups as well as in healthy individuals (Brosschot, Van Dijk, & Thayer, 2007; Patel et al., 2013).

While higher CVC_wake_ and higher CVC_sleep_ have both been linked with better sleep quality, additional research suggests that these links might be due to distinct processes. Some studies did not show a significant relationship between CVC_wake_ and CVC_sleep_ or found that CVC_wake_ – but not CVC_sleep_ – was associated with better sleep quality (Irwin et al., 2006; Jackowska et al., 2012). This might partly be due to variation in CVC_sleep_ across different sleep stages (Stein & Pu, 2012; Tobaldini et al., 2013) and across different periods of the same sleep stage across the night (Snyder, Hobson, Morrison, & Goldfrank, 1964), leading to heterogeneity of CVC_sleep_ during one night of sleep and relatively low reliability across nights. On the other hand, CVC_wake_ has shown good reliability as a stable trait marker (Bertsch, Hagemann, Naumann, Schachinger, & Schulz, 2012) and has been reliably linked to subjective sleep quality.

In summary, evidence suggests that CVC_wake_ and CVC_sleep_ are associated with different processes and are distinct predictors of sleep quality. During wakefulness, CVC corresponds to how people process and flexibly respond to their external environment, which implies the flexible regulation of physiological arousal (Dahl, 1996; Porges, 2007; Thayer & Sternberg, 2006). During sleep, however, CVC is not likely to correspond to online processing and responding to the environment. Rather, CVC_sleep_ may be related to maintaining a generally low arousal state to protect ongoing sleep. Importantly, existing evidence supports CVC_wake_ as the more reliable predictor of sleep quality in comparison to CVC_sleep_.

### 1.3 The present study

The primary aim of the present study was to extend previous research on links between CVC and health by examining the relationship between CVC_wake_ and sleep quality. The present research fills three important gaps. First, prior research has relied predominantly on clinical samples. To advance understanding of the basic relationship between CVC_wake_ and sleep quality, we investigated this question in *healthy adults*. Second, research to date has assessed sleep quality almost exclusively with subjective measures. To thoroughly and validly measure sleep quality, we obtained both *subjective and objective sleep measures*. More specifically, subjective sleep quality was indexed by the Pittsburgh Sleep Quality Index (PSQI; Buysse, Reynolds, Monk, Berman, & Kupfer, 1989) and objective sleep quality by polysomnography (the gold standard for sleep assessments). Third, prior studies assessed CVC_wake_ typically only during relatively short periods (e.g., Fang, Huang, Yang, & Tsai, 2008; Hovland et al., 2013; Kageyama et al., 1998) or waking periods directly before sleep while resting in bed, which can produce confounds with circadian influences (e.g., Irwin et al., 2006; Trinder et al., 2001). In addition, resting baseline assessment without anything to do or focus on may lead to uncontrolled differences in mental activity (Wilson et al., 2014) and reduced reliability. To avoid these limitations, we employed a *minimally-demanding, standardized, and extended baseline* (Jennings, Kamarck, Stewart, Eddy, & Johnson, 1992) in the form of a full-length, emotionally neutral film to assess trait-level CVC_wake_. This baseline assessment approach extends the window for CVC_wake_ assessment to improve its trait-like quality, while concurrently minimizing individual differences in mental activity.

We hypothesized that participants with higher (vs. lower) CVC_wake_, operationalized by heart rate variability in the high-frequency spectral band (0.15–0.40 Hz, HF-HRV, Berntson et al., 1997) would exhibit higher subjective sleep quality (i.e., lower scores on the PSQI) and higher objective sleep quality (assessed by polysomnography). We also assessed HF-HRV_sleep_ across the time spent sleeping and separately for each sleep stage. However, we included these indices primarily for exploratory purposes and, based on the extant literature, we did not expect strong and consistent relationships between HF-HRV_sleep_ and either subjective or objective sleep quality. Finally, we aimed to assess whether associations between HF-HRV_wake_ and sleep quality are specific to sleep quality per se, rather than sleep parameters more broadly construed (e.g., the duration of different sleep stages), which were included in complementary analyses.

## 2 Method

### 2.1 Participants

Participants were 29 healthy female undergraduates (University of Salzburg, all Caucasian) between 19 and 31 years of age (*M* = 23.6 years, SD = 3.3) with a body mass index (BMI) between 17.4 and 31.7 (*M* = 21.8, SD = 3.5). All participants were non- or only occasional smokers with no history of mental, neurological, or sleep disorders. We only accepted participants who were considered in the normal range of subjective sleep quality, as defined by values in the PSQI up to 7 (higher scores indicate poorer sleep) for the past month. The cut-off value for good subjective sleep quality in the PSQI is 5 (Buysse et al., 1989), but values up to 7 are acceptable because this still can be considered within a normal range of sleep quality in female student populations (Pranada, 2005) and we did not want to artificially reduce variance in this variable. The average reported sleep duration on the 3 days before the first night in the laboratory was 8.2 h (SD = 1.1 h).

### 2.2 Procedure

The investigation took place as part of a larger study in the *Clinical Stress and Emotion Lab* of the University of Salzburg. The whole study spanned 11 days. Participants completed daily sleep diaries to assure regular sleep cycles during the whole study. The study included four visits to the lab: the entrance examination, which took place at least 3 days prior to the first night and the 3 nights in the lab each separated by one night, as well as a final 3 days of sleep assessment by sleep diaries. The first night in the lab was used for adaptation and screening purposes (sleep disturbances like sleep apnea, insomnia, periodic leg movements, which did not occur in any of the participants) and was performed two nights prior to the experimental nights. Participants came to the lab around 9 pm and completed several questionnaires including assessment of subjective sleep quality (referring to the prior four weeks) as well as general medical and psychological health condition, e.g., physical fitness (on a 4-point Likert scale, 1 – *not very active* to 4 – *physical activity at least three times a week*) and BMI. After electrodes were attached they went to bed and were woken up after 8 h in bed.

The second and third nights in the laboratory (i.e., night 6 and 8 within the whole study period) were conducted as experimental nights with presentation of a full-length, aversive or emotionally neutral film (counterbalanced order across participants). Results concerning pre-to post-sleep changes in emotional reactivity to the aversive film in relation to REM sleep have been reported separately (Werner, Schabus, Blechert, Kolodyazhniy, & Wilhelm, 2015). The focus of the present investigation was only the neutral film and the subsequent night (either first or second experimental night). On that night, participants were seated on a chair placed 40 inches in front of a 24-inch full HD monitor and the electrodes and sensors for measuring sleep and cardiovascular activity were attached. During film viewing (see below), cardiovascular activity was recorded, whereas during subsequent sleep full polysomnography (PSG, also including cardiovascular activity) was recorded. Stimulus presentation of the film was controlled by E-Prime 2.0 (Psychology Software Tools, Inc., Pittsburgh, PA, USA).

The study was approved by the local ethics committee. Participants signed written informed consent before the study and were compensated with course credit or payment of 100 Euro after the last session of the whole study period.

### 2.3 Measures

#### 2.3.1 HF-HRV

Waking psychophysiological measurements during the neutral film were recorded using a 32-channel amplifier (TMSi, EJ Oldenzaal, The Netherlands) and the recording software package Polybench 1.22 (TMSi) with a sampling rate of 1024 Hz and 22 bit resolution. The recordings contained electrocardiogram (ECG) for which alcohol pads were used to clean the skin sites. ECG was recorded using disposable 55 mm diameter solid-gel snap electrodes; the electrodes were applied on the upper sternum and lowest rib on the left side. The ground electrode was applied at the middle forehead. A 0.05 Hz high-pass filter was applied during ECG measurement. After recording, ECG raw data were bandpass filtered between 0.5 and 40 Hz and further processed using the software Autonomic Nervous System Laboratory (ANSLAB) version 2.51 (Wilhelm, Grossman, & Roth, 1999); R-waves were determined automatically by ANSLAB and further manually checked. The preprocessed ECG was analyzed with power spectral analyses between .15 Hz and .40 Hz. More specifically, to obtain HF-HRV_wake_ values, heart-period time series were linearly detrended and resampled at 4 Hz using cubic spline interpolation. Then, power-spectral densities for the whole duration of the neutral film (94 min) were computed using the Welch algorithm (Welch, 1967), which creates ensemble averages of successive periodograms. The averages were derived from spectra estimated for 120-s segments, overlapping by half. For each 120-s segment, we analyzed 512 points, which includes 480 sampled points with zero padding. We used Hanning-windowed segments that were subjected to fast Fourier transform. Estimates of power were adjusted to account for attenuation produced by the Hanning window and distribution characteristics were normalized by natural-logarithm transformation. We obtained overall HF-HRV_sleep_ values for the time from falling asleep to awakening on the next day (as indicated by PSG analyses) in a similar way as during wakefulness. Furthermore, we obtained HF-HRV_sleep_ for selected sleep stage epochs (≥2 min to increase reliability of spectral analyses and to exclude stage transition periods) and averaged HF-HRV_sleep_ for each sleep stage separately (N1, N2, N3, REM). Although associations between CVC and HF-HRV might be influenced by variables like respiratory rate (e.g., Grossman & Taylor, 2007; Wilhelm, Grossman, & Coyle, 2004), we did not adjust for respiration, as this is less useful for individual difference analyses because participants might differ in respiratory function due to factors unrelated to CVC, such as basal metabolic rate and respiratory pacemaker function (Grossman & Kollai, 1993; Grossman & Taylor, 2007). However, we did control for other possible confounding factors like age, physical fitness, and BMI.

Like others, we used a neutral film to measure HF-HRV_wake_ (Butler, Wilhelm, & Gross, 2006; Jennings et al., 1992; Kogan, Gruber, Shallcross, Ford, & Mauss, 2013). We chose the neutral documentary *Living in a Monastery* (Spiegel TV, 94 min^1^), which describes the daily routine of nuns in a convent without any positive or negative emotional content. The film was validated in a pilot study with 11 female participants (age: *M* = 21.8, SD = 2.0) who provided valence ratings (1 = *positive;* 9 = *negative*) and arousal ratings (1 = *not arousing*, 9 = *very arousing*). Participants rated the film neutral to slightly positive (*M* = 3.4, SD = 1.5) and low arousing (*M* = 2.5, SD = 1.5).

#### 2.3.2 Subjective sleep quality

We used the PSQI (Buysse et al., 1989; German version by Riemann & Backhaus, 1996) to measure subjective sleep quality. The PSQI is a self-report questionnaire assessing sleep quality and sleep disturbances over the preceding 4-week time interval. Eighteen individual items generate seven “component” scores (each with values between 0 and 3): subjective sleep quality, sleep latency, sleep duration, habitual sleep efficiency, sleep disturbances, use of sleeping medication, and daytime dysfunction. These scores are summed up to obtain one global measure of subjective sleep quality (0–21). Lower values in the PSQI indicate better subjective sleep quality.

#### 2.3.3 Objective sleep quality

Night recordings contained full PSG. Electroencephalogram (EEG) and other channels during the night were recorded using a separate 32-channel amplifier (TMSi, EJ Oldenzaal, The Netherlands), which was located next to the bed and using the software package Polybench 1.22 (TMSi). The signals were digitized with a 256 Hz sampling rate and 22 bit resolution. During recording, 0.5 Hz low-pass and 70 Hz high-pass filters were applied. Seven Ag/AgCl electrodes (F3, F4, C3, Cz, C4, O1, O2) were attached with Genuine Grass electrode paste (Grass Technologies, Warwick, US) according to the international 10/20 system with common reference and were later re-referenced to the two additional electrodes on the contralateral mastoids (A1, A2) using Brain Vision Analyzer (Brain Products, Inc., Munich, Germany). Furthermore, four electrooculogram (EOG) channels, two electromyogram (EMG; left and right chin), one bipolar ECG, two bipolar respiratory (thorax and abdomen), one oxygen saturation, and one finger pulse plethysmography channel were recorded. In the screening/adaptation night we used two additional bipolar EMG (musculus tibialis on both sides) and one additional respiratory channel (air flow) to be able to rule out different sleep disorders. Sleep was visually checked and scored by The Siesta Group^©^ (Somnolyzer 24 × 7; c.f., Anderer et al., 2004, 2005) according to the standard criteria of the American Academy of Sleep Medicine (Iber, Ancoli-Israel, Chesson, & Quan, 2007).

Objective sleep quality was indexed by currently accepted measures: total sleep time (TST), sleep efficiency (EFF, defined as TST divided by the time spent in bed in percent), sleep latency (SL, defined as time from going to bed until falling asleep in minutes, measured as stage transition to N2), the wake time after sleep onset, and the number of awakenings during sleep (Blunden & Galland, 2014; Krystal & Edinger, 2008). We also used electroencephalography (EEG) to assess arousals during sleep, which are defined by sudden frequency shifts toward faster rhythms (e.g., theta, alpha, beta, but not spindle activity) that shortly interrupt sleep continuity for at least 3 s (Iber et al., 2007) and are theorized to indicate worse objective sleep quality (Blunden & Galland, 2014; Krystal, Edinger, Wohlgemuth, & Marsh, 2002; Terzano et al., 2003). Sleep architecture variables like the duration of different sleep stages (e.g., N1–N3, rapid eye movement – REM – in minutes) and variables related to specific sleep stages (e.g., REM latency) were included in complementary analyses.

### 2.4 Statistical analysis

We used Pearson correlations to quantify the relationship between HF-HRV_wake_ and indices of sleep quality, i.e., subjective sleep quality, TST, EFF, SL, wake time after sleep onset, number of awakenings during TST as well as number of arousals. In exploratory analyses, we correlated HF-HRV_wake_ with secondary sleep variables related to sleep architecture (sleep stage N1-N3, REM sleep, REM latency). We also examined the relationship of HF-HRV_sleep_ (from falling asleep until awakening on the next morning) and for each sleep stage separately with HF-HRV_wake_ and with primary and secondary sleep variables.

The *α*-level for correlational analyses was set to 0.05. We report effect sizes, Pearson's *r* for correlational analyses or non-parametric Spearman *rho* if normal distribution was not met (*Shapiro–Wilk* < .05). Therefore, we used non-parametric Spearman correlations for the following variables: TST, EFF, SL, wake time after sleep onset, REM latency and BMI. Spearman correlations were also used for ordinally coded variables, i.e., subjective sleep quality (PSQI) and physical fitness. We used partial Spearman correlations when controlling for physical fitness, BMI, and age as simultaneous covariates (Sheskin, 2003). Research often shows inverse links between BMI and physical fitness (e.g., Bovet, Auguste, & Burdette, 2007; Nikolaidis, 2013), which in turn can be linked to sleep quality and HF-HRV. BMI may thus be a reasonable interval-scaled proxy for physical fitness.

## 3 Results

### 3.1 HF-HRV during wakefulness & sleep quality

To investigate the link between HF-HRV_wake_ and sleep quality, we correlated HF-HRV_wake_ with the different indices of sleep quality.

Significant correlations were found for subjective sleep quality as well as for several primary objective variables of sleep quality: Participants with higher levels of HF-HRV_wake_ showed shorter sleep latency and fewer arousals, indicating a less disturbed sleep pattern (see Table 1). TST, EFF, wake time after sleep onset and the number of awakenings were not significantly correlated with HF-HRV_wake_. HF-HRV_wake_ did not correlate significantly with any of the secondary sleep variables, −.13 < *r*s < .18, *p*s > .338.

### 3.2 HF-HRV during sleep & sleep quality

Next, we correlated HF-HRV_sleep_ (on average) with HF-HRV_wake_ as well as with all sleep variables. HF-HRV_sleep_ was significantly related to HF-HRV_wake_, was marginally (but not significantly) related to subjective sleep quality, and was not correlated with any primary (see Table 1) or secondary objective sleep variable, −.16 < *r*s < .26, *p*s > .166. We further correlated HF-HRV_sleep_ for each sleep stage (N1, N2, N3 and REM) with subjective and objective sleep quality. Only higher HF-HRV_sleep_ for N2 and N3 also revealed significant correlations with better subjective sleep quality. However, no significant correlations were found for HF-HRV_sleep_ during sleep stage N1, N2 or N3 with primary variables of objective sleep quality. Higher HF-HRV_sleep_ during REM sleep was related to more awakenings and marginally related to longer wake time after sleep onset (Table 2). Higher HF-HRV_sleep_ during N3 was significantly correlated with one secondary sleep parameter, i.e., shorter duration of sleep stage N1, *r* = −.40, *p* = .032.

### 3.3 Controlling for potential confounds

Finally, we performed partial Spearman correlations with age, physical fitness, and BMI as simultaneous covariates to control for their possible confounding influence. All correlations between HF-HRV_wake_ and subjective sleep quality (i.e., PSQI) as well as objective sleep quality variables (i.e., sleep latency and number of arousals) remained significant, −.54 ≤ *r*s ≤ −.39, *p*s ≤ .049. The marginal correlation between HF-HRV_sleep_ (overall) and subjective sleep quality became non-significant, *r* = −.32, *p* = .108. The correlations between HF-HRV_sleep_ during N2 and N3 with subjective sleep quality became marginal, N2: *r* = −.37, *p* = .062, N3: *r* = −.38, *p* = .058. This was also the case for the correlation between HF-HRV_sleep_ during N3 and sleep stage N1, *r* = −.33, *p* = .096. The correlation between HF-HRV during REM and the number of awakenings became marginal, *r* = .38, *p* = .058, whereas the correlation between HF-HRV_sleep_ during REM and wake time after sleep onset became significant, *r* = .41, *p* = .036.

## 4 Discussion

The primary focus of the present study was the relationship between cardiac vagal control as an individual-difference trait marker, assessed during an extended baseline (CVC_wake_), and both subjective and objective sleep quality in healthy adults. We provide evidence for an association between CVC_wake_ and sleep quality. Specifically, higher HF-HRV_wake_ was associated with better subjective and objective sleep quality (i.e., shorter sleep latency and fewer arousals during sleep) in the subsequent night, whereas this pattern of results was much weaker for HF-HRV_sleep_ (assessed overall and during sleep stages). This suggests that the link with sleep quality is specific to HF-HRV_wake_.

These findings extend the research on clinical populations that demonstrate a link between higher CVC_wake_ and better subjective sleep quality (e.g., Hovland et al., 2013; Irwin et al., 2006; Yang et al., 2011). By assessing healthy adults’ subjective and objective sleep quality, utilizing an optimized assessment design, and controlling for several possible confounds, we believe the present study provides strong evidence for the link between CVC_wake_ – reliably marked by HF-HRV_wake_ – and subjective and objective sleep quality.

Some links were found between HF-HRV_sleep_ (overall, and during N2, N3 and REM sleep) and isolated parameters of sleep quality. Specifically (when controlling for possible confounds), higher HF-HRV_sleep_ during sleep stage N2 and N3 marginally correlated with better subjective sleep quality, but not with primary variables of objective sleep quality. Higher HF-HRV_sleep_ during REM was related to more wake time after sleep onset and marginally related to higher number of awakenings. However, the overall pattern of results was much weaker and less consistent for HF-HRV_sleep_ measures (overall, and for different sleep stages). Since these analyses were mainly included for exploratory purposes without strong empirical or theoretical background they should be considered tentative and replication is important before drawing any conclusions. Nevertheless, the links between HF-HRV_sleep_ (overall, and for N2 and N3) with subjective sleep quality, although mostly marginal, are consistent with earlier studies regarding this topic (Brosschot et al., 2007; Patel et al., 2013).

### 4.1 Theoretical implications

The present results converge with existing research to suggest that CVC_wake_ is related to the flexible regulation of arousal, which has important implications for its role in supporting high-quality sleep. Beyond implications for the role of CVC_wake_ in sleep, the present results have implications for how CVC_wake_ is conceptualized more generally.

#### 4.1.1 Implications for understanding the role of CVC in sleep quality

Our results are characterized by a pattern that supports CVC_wake_ as a key marker of arousal-related processes. Specifically, and consistent with prior research using actigraphy (Elmore-Staton et al., 2012; Palesh et al., 2008), we assessed a variety of sleep variables and found that CVC_wake_ was only related to variables assessing sleep quality (e.g., sleep latency, number of arousals during sleep) and not variables that are more related to sleep quantity or sleep architecture (e.g., total sleep time, amount of N1-N3, REM, REM latency) associated with specific cognitive processes during sleep like, for example, memory consolidation (e.g., Ackermann & Rasch, 2014; Diekelmann, Wilhelm, & Born, 2009).

Higher CVC_wake_ was associated with shorter sleep latency. The transition from wake to sleep is thought to be accompanied by a simultaneous reduction in autonomic arousal (Trinder et al., 2001) and high CVC_wake_ may accelerate autonomic regulation toward this transition. On the other hand, arousals during sleep are related to sleep disruptions due to interspersed short periods of higher EEG spectral frequencies more characteristic of wake states (Iber et al., 2007). Consequently, the link between higher CVC_wake_ and better sleep quality seems to specifically reflect the ability to reduce autonomic arousal in the anticipation of sleep as well as central arousal during sleep. In other words, lower CVC_wake_ related to higher autonomic arousal or perhaps the inhibition of de-arousal processes (see Espie, 2002; Winzeler et al., 2014) may impair sleep quality by lengthening sleep latency and disrupting sleep continuity, while variables more related to sleep architecture are left largely unchanged (e.g., Bonnet & Arand, 2003; Carskadon et al., 1976).

The specific relationship between CVC_wake_ and sleep variables related to arousal might further explain why our results do not support a link between CVC_wake_ and all variables we chose for measuring sleep quality. Krystal and Edinger (2008) address the problematic lack of an established definition for objective sleep quality and point out that sleep quality can refer to various polysomnographically measured sleep variables (see also Buysse, Ancoli-Israel, Edinger, Lichstein, & Morin, 2006). However, they emphasize other results showing that some individuals report sleep complaints while these objective sleep variables are comparable to individuals without sleep complaints (e.g., Carskadon et al., 1976; Coleman et al., 1982). Our results underscore the relationship between CVC_wake_ and specific variables of sleep quality and suggest that total sleep time or the number of awakenings might be related to sleep processes other than arousal regulation (see Krystal & Edinger, 2008).

#### 4.1.2 Implications for understanding CVC

Our results converge with several lines of evidence to suggest that it is CVC_wake_ that is linked to the flexible regulation of arousal. We found that only CVC_wake_, but not CVC_sleep_ was related to objective sleep quality, although all indices of CVC were highly correlated. The current results highlight the importance of CVC assessed during wakefulness (when the organism is required to interact with its environment) compared to CVC assessed during sleep (when the organism is unable to interact with its environment). Specifically, other studies that have assessed both CVC_wake_ and CVC_sleep_ have also found that only CVC_wake_ is linked with sleep quality, at least when assessing non-healthy adult samples (e.g., Irwin et al., 2006). Using objective measures of sleep quality in healthy adults, the present results extend these findings and suggest that CVC_wake_ may carry more predictive power than CVC_sleep_ (overall or during specific sleep stages), when using sleep quality as an outcome of interest. This argument is even more salient when considering that CVC_wake_ measurement was separated in time from the objective sleep measures, while CVC_sleep_ measurement was obtained in parallel with objective sleep measures.

The link between CVC_wake_ and the flexible regulation of autonomic arousal is also consistent with several models supporting CVC_wake_ as important predictor for health-related outcomes more generally. First, conceptual models support CVC_wake_ as a marker of arousal-related processes especially in the context of perceived stress (e.g., Porges, 2007). Evolutionarily, it is important to attend to the environment and to be able to respond with a “fight or flight” reaction. Whereas the actual reaction is related to decreased CVC_wake_ (often called withdrawal of vagal activity or vagal reactivity) and enhanced autonomic arousal, the state of surveying the environment is related to increased CVC_wake_ and reduced autonomic arousal. Importantly, this vigilance function is not necessary while the organism is asleep, a state that is restricted to times and places when the organism is relatively safe (Dahl, 1996). Second, another theory of CVC_wake_ has emphasized its role as a marker of self-regulation and flexible adaptation to changing physiological and environmental demands (Thayer & Lane, 2000, 2009). This model of neurovisceral integration assumes that CVC_wake_ is related to a system that continuously evaluates the environment for different signs of danger and safety and prepares the organism for appropriate reactions, and promoting general adaptability to the environment. Thus, CVC assessed while the organism is awake and able to interact effectively with the environment may be more predictive of that organism's underlying self-regulation abilities than CVC assessed during sleep.

Together, these lines of evidence converge to support a model in which CVC_wake_ is a marker of processes related to the flexible regulation of autonomic arousal. We propose this as an underlying factor for why CVC_wake_ is related to effective sleep regulation, but also attentional and emotional processes, as well as various physical and mental health conditions related to well-regulated autonomic arousal.

### 4.2 Limitations and future directions

The present research suggests that higher CVC_wake_ is related to better subjective and objective sleep quality. We acknowledge four possible limitations that suggest important future directions. First, because sleep is known to vary across the lifespan and between genders (e.g., Carrier, Land, Buysse, Kupfer, & Monk, 2001; Dijk, Beersma, & Bloem, 1989; Tsai & Li, 2004), our research involved only young women. This might limit the generalizability of the present study, and future research should investigate the relationship between CVC and sleep quality also in healthy men and older populations. Second, the use of a neutral film to assess CVC_wake_ could have elicited an orienting response. However, we believe an orienting response would be more prominent early in the film, as we would predict a habituation response over the course of the 94-min neutral documentary. Additionally, this similarly would have affected all participants and should not have created a selective bias in individual difference correlational analyses. In relation to that, one could argue that this response during the neutral film might reflect a state of enhanced vigilance or automatic processing of visual and auditory stimuli. However, as described in the introduction, others suggest that this baseline assessment is equivalent to a resting baseline (concerning cardiovascular measures), but reveals greater stability and is better able to create a neutral context (e.g., Jennings et al., 1992; Wilson et al., 2014). Third, as we used a correlational design, we are not able to draw causal conclusions. Higher levels of CVC_wake_ may promote higher sleep quality. Prior research has demonstrated that experimental manipulation of CVC_wake_ by biofeedback training improved health (e.g., Nolan et al., 2005). However, sleep clearly influences cardiovascular functioning (e.g., Gangwisch et al., 2006) and thus CVC_wake_ and sleep quality are likely linked in a bidirectional fashion. Future research could use experimental designs or longitudinal assessments to examine more directly the causal direction between CVC_wake_ and sleep quality. Finally, we investigated the relationship between CVC_wake_ and sleep quality as one important marker of health. Other research has examined and reliably linked CVC_wake_ with other components of physical and mental health such as cardiovascular disease, depression, or psychological well-being (e.g., Beauchaine, 2001; Rottenberg, Clift, Bolden, & Salomon, 2007; Thayer & Lane, 2007). There are two plausible models how CVC_wake_ and sleep quality might be linked to health more broadly. On the one hand, sleep may mediate the association between CVC_wake_ and different health outcomes. On the other hand, research linking both CVC_wake_ and sleep to, e.g., psychological health outcomes suggest that high sleep quality and high CVC_wake_ might interact to predict health (e.g., El-Sheikh et al., 2007). Future research should test these different models to better understand how CVC_wake_ and sleep quality interact to shape psychological and physical health.

## 5 Concluding comments

To our knowledge, this is the first investigation to link individual differences in CVC_wake_ to different variables of objective sleep quality in healthy adults. Results showed that higher CVC_wake_ is related to better subjective and objective sleep quality, whereas the pattern of results is much weaker for CVC_sleep_. Thus, high levels of CVC_wake_ may be one particularly important ingredient of healthy sleep.

## Funding

The first author of this paper was financially supported by the Doctoral College “Imaging the Mind” of the Austrian Science Fund (FWF-W1233). The second author of this paper was financially supported by a National Institutes of Health Affective Science Training Fellowship (5T32MH020006).

## Figures and Tables

**Table 1:** Descriptive statistics and correlations for HF-HRV_wake_ and HF-HRV_sleep_ (whole night) with primary indices of sleep quality.

Variables	*M*	SD	HF-HRV_wake_	HF-HRV_sleep_
HF-HRV_wake_ (ms^2^)^a^	8.17	1.02	−	
HF-HRV_sleep_ (ms^2^)^a^	8.77	0.80	.52**	−
PSQI (sum score)^b^	4.10	1.37	−.39*	**−.32**+
Sleep latency (min)^b^	25.71	19.06	−.43*	−.17
Total sleep time (min)^b^	445.81	22.97	.06	−.17
Sleep efficiency (%)^b^	92.86	4.70	.07	−.18
Wake after sleep onset (min)^b^	13.50	15.81	.25	.26
Awakenings (*N*)^a^	10.03	6.29	.19	.24
Arousals (*N*)^a^	177.62	67.47	−.53**	−.03

*Note*: *N* = 29. HF-HRV = high-frequency heart rate variability; PSQI = Pittsburgh Sleep Quality Index, with lower values indicating better sleep quality. Correlation between: HF-HRV wake & PSQI: p = .039; HF-HRV wake & SL: p = .022; HF-HRV wake & Arousals: p = .003; HF-HRV sleep & PSQI: p = .086.

a Pearson correlations.

b Spearman rho correlations.

* *p* < .05.

** *p* < .01.

+ *p* < .10.

**Table 2:** Descriptive statistics and correlations for HF-HRV_sleep_ during each sleep stage separately with primary indices of sleep quality.

Variables	*M*	SD	HF-HRV N1	HF-HRV N2	HF-HRV N3	HF-HRV REM
HF-HRV N1 (ms^2^)^a^	8.73	0.97	–			
HF-HRV N2 (ms^2^)^a^	8.57	0.92	.85**	–		
HF-HRV N3 (ms^2^)^a^	8.37	1.01	.79**	.93**	–	
HF-HRV REM (ms^2^)^a^	8.53	0.97	.81**	.83**	.69**	–
PSQI (sum score)^b^	4.10	1.37	−.23	−.37*	−.38*	−.26
Sleep latency (min)^b^	25.71	19.06	−.22	−.17	−.10	−.13
Total sleep time (min)^b^	445.81	22.97	−.10	−.11	−.01	−.23
Sleep efficiency (%)^b^	92.86	4.70	−.10	−.12	−.04	−.23
Wake after sleep onset (min)^b^	13.50	15.81	.29	.20	−.01	**.36**+
Awakenings (*N*)^a^	10.03	6.29	.25	.21	.09	.37*
Arousals (*N*)^a^	177.62	67.47	−.15	−.13	−.16	.00

*Note*: *N* = 29. For HF-HRV N1 *N* = 28, because no N1 segment was longer than 2 min in one participant. HF-HRV = high-frequency heart rate variability; PSQI = Pittsburgh Sleep Quality Index, with lower values indicating better sleep quality; HF-HRV N1/N2/N3/REM: HF-HRV during different sleep stages. Correlation between: HF-HRV N2 &PSQI: p = .035; HF-HRV N3 & PSQI: p = .029; HF-HRV REM & Wake after sleep onset: p = .055; HF-HRV REM & Awakenings: p = .049.

a Pearson correlations.

b Spearman rho correlations.

* *p* < .05.

** *p* < .01.

+ *p* < .10.

## Footnotes

1 This German documentary can be found on the webpage of the television channel *SpiegelTV* (http://www.spiegel.tv/filme/kloster-leben-verliebt-gott/).
